# Regions of High Out-Of-Hospital Cardiac Arrest Incidence and Low Bystander CPR Rates in Victoria, Australia

**DOI:** 10.1371/journal.pone.0139776

**Published:** 2015-10-08

**Authors:** Lahn D. Straney, Janet E. Bray, Ben Beck, Judith Finn, Stephen Bernard, Kylie Dyson, Marijana Lijovic, Karen Smith

**Affiliations:** 1 Department of Epidemiology and Preventive Medicine, Monash University, Melbourne, Victoria, Australia; 2 Faculty of Health Science, Curtin University, Perth, Western Australia, Australia; 3 St John Ambulance Western Australia, Perth, Western Australia, Australia; 4 Intensive Care Unit, The Alfred Hospital Melbourne, Victoria, Australia; 5 Ambulance Victoria, Melbourne, Victoria, Australia; Azienda Ospedaliero-Universitaria Careggi, ITALY

## Abstract

**Background:**

Out-of-hospital cardiac arrest (OHCA) remains a major public health issue and research has shown that large regional variation in outcomes exists. Of the interventions associated with survival, the provision of bystander CPR is one of the most important modifiable factors. The aim of this study is to identify census areas with high incidence of OHCA and low rates of bystander CPR in Victoria, Australia

**Methods:**

We conducted an observational study using prospectively collected population-based OHCA data from the state of Victoria in Australia. Using ArcGIS (ArcMap 10.0), we linked the location of the arrest using the dispatch coordinates (longitude and latitude) to Victorian Local Government Areas (LGAs). We used Bayesian hierarchical models with random effects on each LGA to provide shrunken estimates of the rates of bystander CPR and the incidence rates.

**Results:**

Over the study period there were 31,019 adult OHCA attended, of which 21,436 (69.1%) cases were of presumed cardiac etiology. Significant variation in the incidence of OHCA among LGAs was observed. There was a 3 fold difference in the incidence rate between the lowest and highest LGAs, ranging from 38.5 to 115.1 cases per 100,000 person-years. The overall rate of bystander CPR for bystander witnessed OHCAs was 62.4%, with the rate increasing from 56.4% in 2008–2010 to 68.6% in 2010–2013. There was a 25.1% absolute difference in bystander CPR rates between the highest and lowest LGAs.

**Conclusion:**

Significant regional variation in OHCA incidence and bystander CPR rates exists throughout Victoria. Regions with high incidence and low bystander CPR participation can be identified and would make suitable targets for interventions to improve CPR participation rates.

## Introduction

Out-of-hospital cardiac arrest (OHCA) remains a major public health issue [1]. The high case fatality rate of OHCA (>90%) in most communities[2,3], indicates the importance of developing appropriate interventional strategies [4].

Efforts to improve out-of-hospital cardiac arrest outcomes focus on improving the chain of survival [5,6]. This includes early recognition of cardiac arrest symptoms, early CPR, early defibrillation and early advanced post-resuscitation care [7]. Of the interventions associated with survival, the provision of bystander CPR is one of the most important modifiable factors[8–14]. Improvements in bystander CPR rates through system changes, such as simplification of CPR instructions in the emergency call, have been reported[15]. However, whole-of-community interventions have shown mixed results[16], suggesting that such interventions might be better placed in communities with a high incidence of OHCA and a low prevalence of bystander CPR.

Research in the US has shown that these ‘high-risk’ census tracts can be identified by geocoding OHCA registry data[17,18]. However, it is unknown if such high-risk areas exist in the Australian community and whether these communities remain at constant high-risk over time. Therefore, the aim of this study is to identify census areas with high incidence of OHCA and low rates of bystander CPR in Victoria, Australia. In addition, this study aims to evaluate changes in these measures over time.

## Methods

### Study Design and Setting

We conducted an observational study using prospectively collected population-based OHCA data from the state of Victoria in Australia. Victoria has a current population of 5.6 million, 75% of whom reside in the metropolitan region of Melbourne. Ambulance Victoria (AV) is the sole provider of Emergency Medical Services (EMS) in the state. AV delivers a two-tiered EMS system, with Advanced Life Support Paramedics and Intensive Care Ambulance Paramedics. Fire fighters and volunteer Community Emergency Response Teams provide a first response in select areas of Victoria.

### The Victorian Ambulance Cardiac Arrest Registry (VACAR)

AV maintains the Victorian Ambulance Cardiac Arrest Registry (VACAR), which registers and collects EMS clinical and outcome data for all OHCA attended by EMS in the state of Victoria[19]. Data collection is standardized using the Utstein definitions[20]. The Victorian Department of Health Human Research Ethics Committee (HREC) has approved VACAR (No. 08/02) data collection. Ethics approval for the current study was received from the Monash University Human Research Ethics Committee (CF12/3410–2012001638).

### Inclusion and Exclusion Criteria

The VACAR was searched and data was extracted for OHCA cases occurring between January 2008 and December 2013. To match with available population data, cases were included if they were aged greater than 20 years and the arrest was presumed to be of cardiac etiology based on EMS documentation (i.e. no other obvious cause recorded such as trauma, hanging, drowning, etc.).

### Local Government Areas (LGAs) and Geospatial Mapping

Australia has a federal system of government under which state governments preside in each of the eight states and territories. Beneath this are local governments, for which there are 79 local government areas (LGAs) in the state of Victoria.

Using ArcGIS (ArcMap 10.0), we linked the location of the arrest using the dispatch coordinates (longitude and latitude) to Victorian LGAs.

### Statistical Analysis

We restricted the calculation of bystander CPR rates to those arrests that were witnessed by a bystander (not a paramedic). We coded cases in which the patient received bystander chest compressions, even if ‘stated as inadequate or poor’, as having received bystander CPR. We defined the absence of bystander CPR as those cases that received no bystander chest compressions, or who received ventilation only. We excluded cases that were coded as ‘unknown’ or ‘not stated’ from estimates of bystander CPR rates (259, 4.1%).

We calculated the incidence of OHCA in Victorian LGAs using yearly population data from the Australian Bureau of Statistics, and bystander CPR rates for each LGA[21]. We used Bayesian hierarchical models with random effects on each LGA to provide shrunken estimates of the rates of bystander CPR and the incidence rates. We adjusted for relative socio-economic advantage and disadvantage using the 2011 Socio-Economic Indexes for Areas, ‘SEIFA index’, for each LGA[22]. The rates were modelled using a mixed-effects logit model for bystander CPR and a mixed-effects Poisson model for the OHCA incidence rate, where the random effect was assumed to be normally distributed with a mean of zero. These models estimate the ‘shrunken’ LGA rate as a weighted average of the pooled estimate (adjusted by SEIFA index) and the LGA-specific estimate [23]. The weights that contribute to this average are the inverse variances of the pooled estimate and the LGA-specific estimate. Thus an LGA with considerably fewer OHCA events will shrink toward the pooled estimate more than an LGA with many observed events. In this way, those LGAs with very few events are shrunken considerably toward the pooled estimate (or adjusted mean) regardless of their observed rates.

We considered two time periods to examine changes over time, 2008–2010 versus 2011–2013. These time periods also correspond with the introduction of the 2010 ILCOR guidelines. We plotted the shrunken estimates of the rates of bystander CPR and the incidence of OHCA in each LGA.

## Results

Over the study period there were 31,019 adult OHCA attended, of which 21,436 (69.1%) cases were of presumed cardiac etiology. For those cases of presumed cardiac etiology, the overall incidence of OHCA was 64.6 cases per 100,000 person-years. The crude incidence declined from 66.6 cases per 100,000 person-years in 2008–2010 to 62.6 cases per 100,000 person-years in 2011–2013 (p<0.001).

The overall rate of bystander CPR for bystander witnessed OHCAs was 62.4%, with the rate increasing from 56.4% in 2008–2010 to 68.6% in 2010–2013.

### Regional OHCA Incidence

Among LGAs, the median crude annual incidence rate of OHCA was 71.3 cases per 100,000 person-years (IQR: 58.5–79.0).

**Fig 1:**
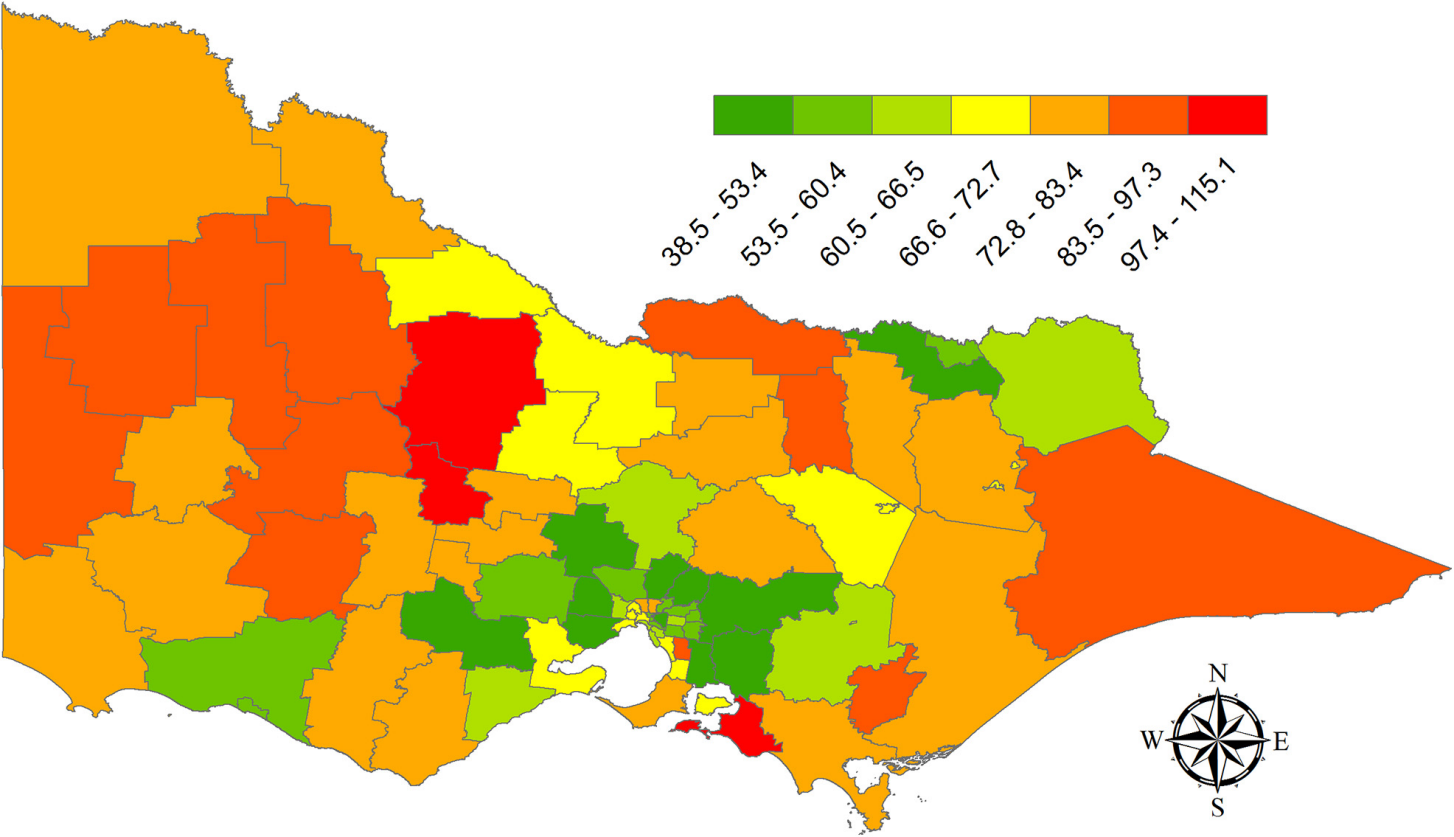
Shrunken estimate of OHCA incidence rate (per 100,000 person years) for Victorian Local Government Areas 2008–2013.

The map shown in Fig 1 provides the shrunken estimate of the incidence in each LGA. Significant variation among LGAs was observed with a 3 fold difference in the incidence rate between the lowest and highest LGAs. The highest incidence rates were seen in two neighboring rural LGAs (Central Goldfields LGA = 115.1 cases per 100,000 person-years; Loddon LGA = 110.6 cases per 100,000 person-years). The lowest incidence rates were generally in outer suburban areas surrounding Melbourne, with the lowest incidence rate found in the North-East of Melbourne in the Shire of Nillumbik (38.5 cases per 100,000 person-years).

### Regional Bystander CPR

**Fig 2:**
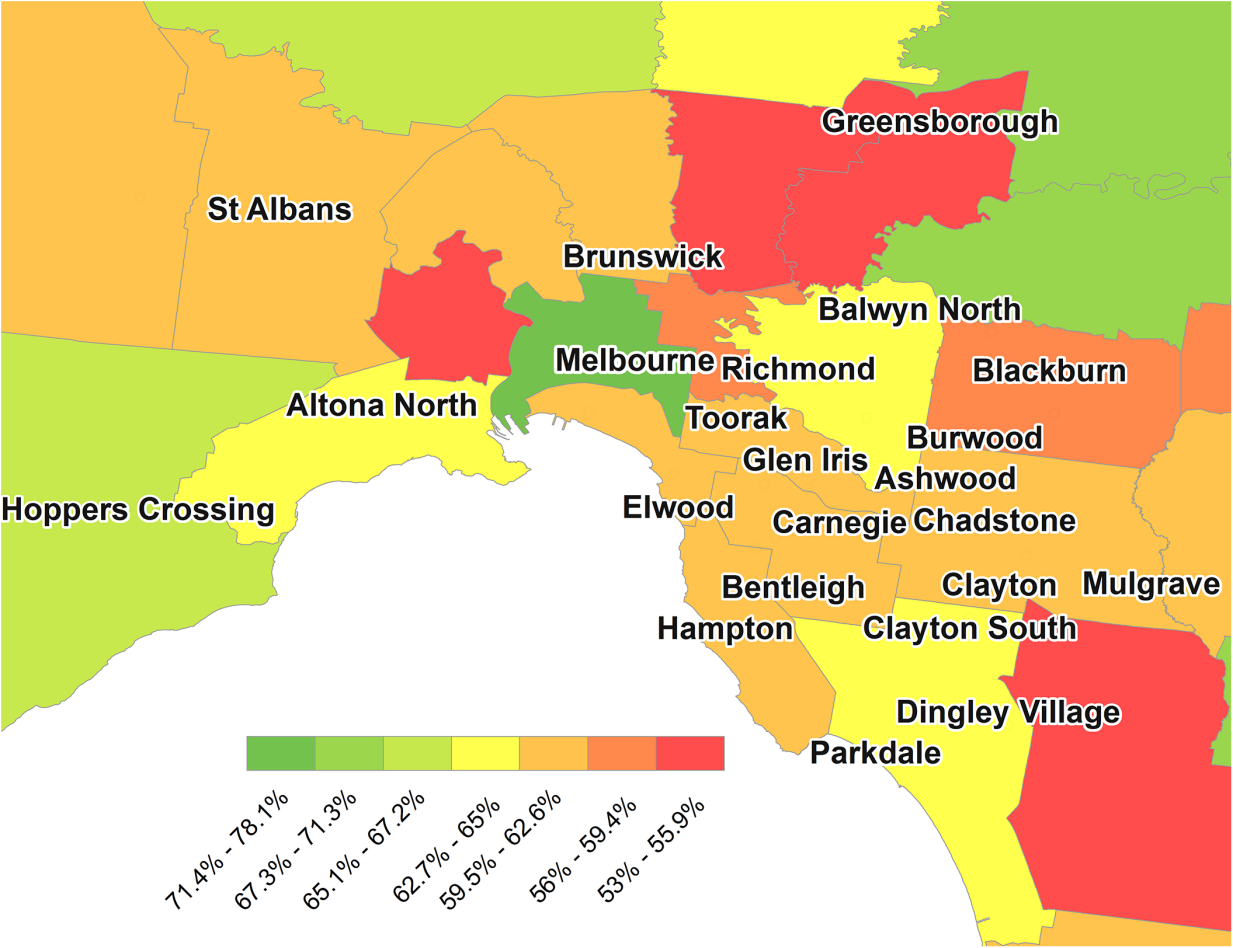
Prevalence of Bystander CPR in Melbourne and surrounding areas, 2008–2013 by Victorian Local Government Area.

The median estimate of bystander CPR among LGAs was 63.2% (IQR: 60.5%-65.7%) over the entire study period. Fig 2 provides a map of Melbourne City and surrounding local government areas. Even across the metropolitan regions, large disparities in the rate of bystander CPR can be seen. There was a 25.1% absolute difference in bystander CPR rates between the highest and lowest LGAs. The highest rate of bystander CPR was in Melbourne city with a rate of 78.1%. In contrast the rate of bystander CPR was lowest in Greater Dandenong, South-East of Melbourne with a rate of 53.0%.

**Fig 3:**
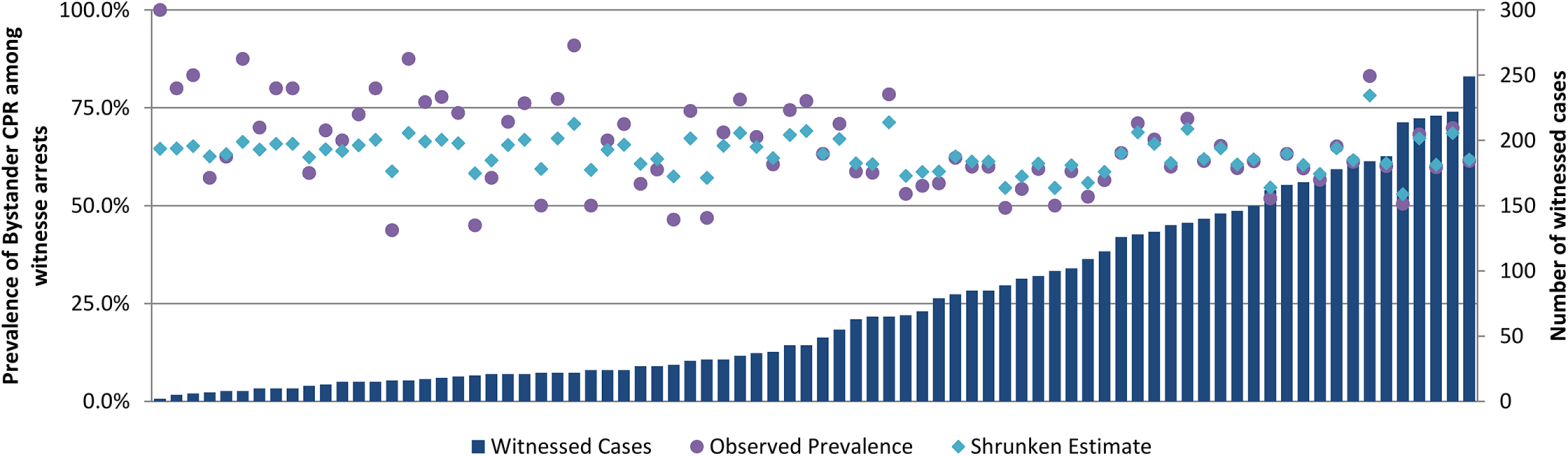
Bystander witnessed OHCA cases by Victorian Local Government Area with the Observed Prevalence and shrunken estimate of Bystander CPR among witnessed arrests, 2008–2013.

Fig 3 provides the observed OHCA events, the crude rate of bystander CPR and the shrunken estimate in each LGA during the study period. While crude rates varied from 43.8% to 100%, these were in LGAs with a very small number of OHCA events.

**Fig 4:**
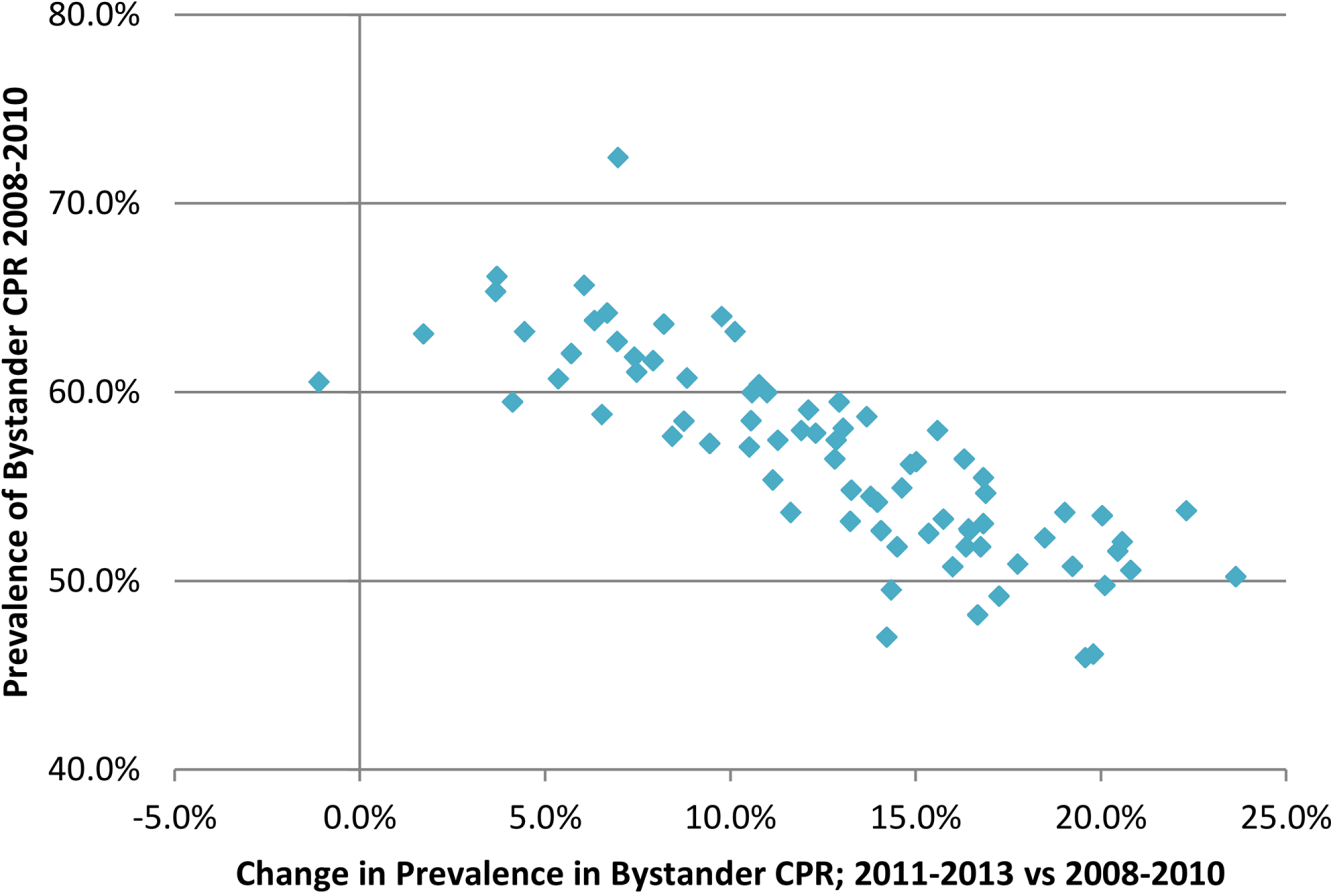
Prevalence of Bystander CPR in 2008–2010, and change between 2008–2010 and 2011–2013 by Victorian Local Government Area.

In all but one LGA the rates of bystander CPR improved during the study period. Fig 4 shows the change in the estimated rate from 2008–2010 to 2011–2013. The largest improvements in bystander CPR occurred in the LGAs with the lowest rates in 2008–2010. (p<0.001)

**Fig 5:**
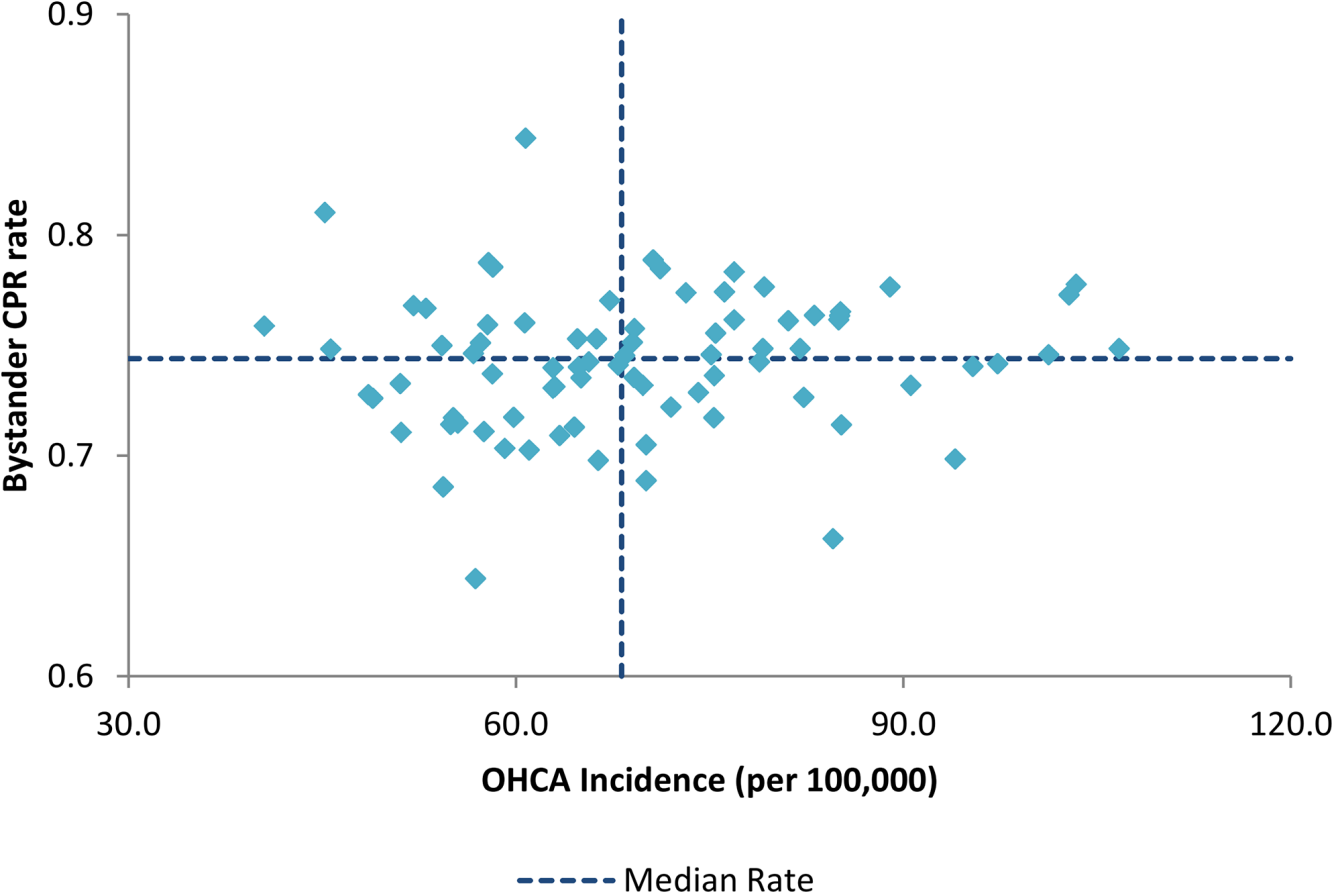
Incidence of OHCA and the prevalence of Bystander CPR 2011–2013 by Victorian Local Government Area.

Fig 5 shows the incidence of OHCA against the prevalence of bystander CPR for the period 2011–2013 in each LGA. The lower right quadrant indicates those regions with an incidence rate higher than the median with bystander CPR rates that are lower than the median.

Tables 1 and 2 provide a complete list of the incidence (Table 1) and bystander CPR rates (Table 2) for each LGA for the entire period, as well as split into the first half and second half of the study period.

**Table 1:** Shrunken estimate of Out-of Hospital Cardiac Arrest incidence rate by Victorian Local Government Area, 2008–2013.

		Incidence Rate (shrunken estimate per 100,000 person years)			2013 Population over 20 years
LGA Name	LGA Code	2008–2010	2010–2013	2008–2013	
Alpine (S)	20110	80.4	75.5	80.3	12 044
Ararat (RC)	20260	82.3	101.2	97.3	11 207
Ballarat (C)	20570	85.2	67.9	76.6	98 684
Banyule (C)	20660	56.7	63.4	60.2	124 475
Bass Coast (S)	20740	103.9	97.3	105.5	31 010
Baw Baw (S)	20830	64.1	62.9	62.5	45 205
Bayside (C)	20910	65.1	55.2	61.1	98 368
Benalla (RC)	21010	92.5	79.1	88.5	13 719
Boroondara (C)	21110	56.2	48.6	52.6	170 553
Brimbank (C)	21180	71.2	63.0	66.5	195 469
Buloke (S)	21270	82.4	85.1	88.5	6 221
Campaspe (S)	21370	77.9	67.3	71.8	36 919
Cardinia (S)	21450	47.5	57.9	51.7	84 065
Casey (C)	21610	49.1	45.7	46.6	275 116
Central Goldfields (S)	21670	111.1	106.7	115.1	12 602
Colac-Otway (S)	21750	69.9	82.3	76.1	20 694
Corangamite (S)	21830	70.4	76.9	74.4	16 137
Darebin (C)	21890	80.4	70.1	75.5	146 797
East Gippsland (S)	22110	92.3	85.2	90.3	43 413
Frankston (C)	22170	74.2	68.4	71.4	133 560
Gannawarra (S)	22250	67.0	81.1	72.7	10 326
Glen Eira (C)	22310	67.2	54.4	61.1	141 519
Glenelg (S)	22410	74.4	82.0	78.6	19 521
Golden Plains (S)	22490	57.3	54.3	52.9	20 151
Greater Bendigo (C)	22620	71.9	69.1	70.4	105 332
Greater Dandenong (C)	22670	87.9	84.5	86.2	146 727
Greater Geelong (C)	22750	68.6	70.6	69.6	221 515
Greater Shepparton (C)	22830	75.8	75.3	75.4	62 784
Hepburn (S)	22910	68.0	79.2	74.4	14 843
Hindmarsh (S)	22980	83.7	95.4	95.3	5 695
Hobsons Bay (C)	23110	77.5	66.4	72.3	89 111
Horsham (RC)	23190	78.0	69.9	74.4	19 687
Hume (C)	23270	64.7	57.2	60.1	183 263
Indigo (S)	23350	55.1	58.2	53.2	15 372
Kingston (C)	23430	72.0	65.0	68.9	151 686
Knox (C)	23670	51.7	59.1	55.2	154 909
Latrobe (C)	23810	88.2	94.0	92.1	73 846
Loddon (S)	23940	98.4	102.8	110.6	7 443
Macedon Ranges (S)	24130	48.9	58.2	52.9	44 098
Manningham (C)	24210	55.8	52.1	54.0	117 537
Mansfield (S)	24250	76.2	65.7	72.0	8 185
Maribyrnong (C)	24330	71.5	70.1	71.0	79 302
Maroondah (C)	24410	59.9	55.0	57.1	109 575
Melbourne (C)	24600	77.7	60.7	69.5	116 447
Melton (S)	24650	46.6	48.9	46.2	122 909
Mildura (RC)	24780	70.6	83.1	76.4	52 685
Mitchell (S)	24850	61.0	64.5	61.5	37 366
Moira (S)	24900	92.7	89.0	93.2	28 675
Monash (C)	24970	58.6	55.5	57.0	182 485
Moonee Valley (C)	25060	74.5	64.8	70.2	115 097
Moorabool (S)	25150	67.4	56.7	60.4	30 320
Moreland (C)	25250	78.6	69.2	74.1	160 029
Mornington Peninsula (S)	25340	76.7	74.1	76.0	152 260
Mount Alexander (S)	25430	76.9	75.4	77.5	17 994
Moyne (S)	25490	63.3	57.8	58.7	16 277
Murrindindi (S)	25620	72.8	75.1	75.8	13 494
Nillumbik (S)	25710	39.8	40.5	38.5	62 724
Northern Grampians (S)	25810	80.6	103.4	96.4	11 799
Port Phillip (C)	25900	65.9	57.5	62.3	102 501
Pyrenees (S)	25990	67.5	90.6	78.9	6 770
Queenscliffe (B)	26080	69.5	66.2	76.0	3 058
South Gippsland (S)	26170	77.4	71.2	75.2	27 930
Southern Grampians (S)	26260	76.1	76.9	78.6	16 145
Stonnington (C)	26350	61.9	51.1	56.9	103 187
Strathbogie (S)	26430	75.8	85.0	83.4	9 706
Surf Coast (S)	26490	59.8	60.7	61.5	28 282
Swan Hill (RC)	26610	73.6	76.2	74.1	20 867
Towong (S)	26670	65.1	69.0	66.0	5 889
Unincorporated Vic	29399	66.0	64.7	67.8	758
Wangaratta (RC)	26700	79.0	72.0	76.0	27 197
Warrnambool (C)	26730	57.1	62.9	57.7	33 300
Wellington (S)	26810	77.7	73.2	75.7	42 319
West Wimmera (S)	26890	79.0	78.8	85.0	4 089
Whitehorse (C)	26980	69.6	61.0	65.7	161 724
Whittlesea (C)	27070	54.6	51.1	51.8	179 261
Wodonga (RC)	27170	64.4	59.8	59.9	37 345
Wyndham (C)	27260	48.0	45.2	45.4	189 618
Yarra (C)	27350	61.2	56.9	58.9	83 593
Yarra Ranges (S)	27450	54.9	53.0	53.4	149 538
Yarriambiack (S)	27630	80.9	85.1	86.2	7 018

Cities (C), Rural Cities (RC), Boroughs (B) and Shires (S)

**Table 2:** Shrunken estimate of bystander CPR rate by Victorian Local Government Area, 2008–2013.

		Bystander CPR (Shrunken proportion of bystander witnessed arrests due to presumed cardiac aetiology)			2013 Population over 20 years
LGA Name	LGA Code	2008–2010	2010–2013	2008–2013	
Alpine (S)	20110	60.0%	70.5%	65.9%	12 044
Ararat (RC)	20260	61.7%	69.6%	66.4%	11 207
Ballarat (C)	20570	52.7%	69.1%	60.4%	98 684
Banyule (C)	20660	46.1%	65.9%	55.9%	124 475
Bass Coast (S)	20740	52.7%	69.2%	60.6%	31 010
Baw Baw (S)	20830	65.3%	69.0%	68.1%	45 205
Bayside (C)	20910	57.3%	66.7%	61.3%	98 368
Benalla (RC)	21010	53.0%	69.9%	61.6%	13 719
Boroondara (C)	21110	62.1%	67.8%	64.7%	170 553
Brimbank (C)	21180	54.2%	68.1%	60.5%	195 469
Buloke (S)	21270	56.3%	71.3%	65.8%	6 221
Campaspe (S)	21370	51.6%	72.0%	62.1%	36 919
Cardinia (S)	21450	64.0%	73.8%	71.3%	84 065
Casey (C)	21610	66.1%	69.8%	68.5%	275 116
Central Goldfields (S)	21670	49.8%	69.9%	59.2%	12 602
Colac-Otway (S)	21750	63.2%	67.6%	65.6%	20 694
Corangamite (S)	21830	60.4%	71.2%	67.2%	16 137
Darebin (C)	21890	49.5%	63.9%	54.7%	146 797
East Gippsland (S)	22110	53.2%	66.4%	58.8%	43 413
Frankston (C)	22170	54.9%	69.5%	61.9%	133 560
Gannawarra (S)	22250	58.1%	71.1%	65.8%	10 326
Glen Eira (C)	22310	59.5%	63.6%	60.6%	141 519
Glenelg (S)	22410	58.0%	69.9%	64.3%	19 521
Golden Plains (S)	22490	50.8%	70.0%	59.4%	20 151
Greater Bendigo (C)	22620	51.8%	68.6%	58.7%	105 332
Greater Dandenong (C)	22670	47.0%	61.2%	53.0%	146 727
Greater Geelong (C)	22750	50.2%	73.9%	61.8%	221 515
Greater Shepparton (C)	22830	52.7%	66.7%	58.7%	62 784
Hepburn (S)	22910	52.1%	72.6%	64.0%	14 843
Hindmarsh (S)	22980	58.5%	69.0%	64.4%	5 695
Hobsons Bay (C)	23110	63.1%	64.8%	63.4%	89 111
Horsham (RC)	23190	51.8%	68.2%	58.3%	19 687
Hume (C)	23270	63.8%	70.1%	67.2%	183 263
Indigo (S)	23350	57.4%	68.7%	63.2%	15 372
Kingston (C)	23430	61.1%	68.5%	64.7%	151 686
Knox (C)	23670	58.8%	65.3%	61.8%	154 909
Latrobe (C)	23810	48.2%	64.9%	54.6%	73 846
Loddon (S)	23940	55.5%	72.3%	65.4%	7 443
Macedon Ranges (S)	24130	58.0%	73.5%	68.6%	44 098
Manningham (C)	24210	63.6%	71.8%	68.7%	117 537
Mansfield (S)	24250	56.4%	69.3%	62.4%	8 185
Maribyrnong (C)	24330	45.9%	65.5%	54.6%	79 302
Maroondah (C)	24410	49.2%	66.4%	57.5%	109 575
Melbourne (C)	24600	72.4%	79.4%	78.1%	116 447
Melton (S)	24650	57.1%	67.6%	62.6%	122 909
Mildura (RC)	24780	50.5%	71.4%	60.8%	52 685
Mitchell (S)	24850	51.8%	66.3%	57.5%	37 366
Moira (S)	24900	53.6%	72.7%	63.3%	28 675
Monash (C)	24970	55.3%	66.5%	60.4%	182 485
Moonee Valley (C)	25060	53.3%	69.0%	60.9%	115 097
Moorabool (S)	25150	62.7%	69.6%	67.2%	30 320
Moreland (C)	25250	52.3%	70.8%	61.7%	160 029
Mornington Peninsula (S)	25340	52.5%	67.9%	60.8%	152 260
Mount Alexander (S)	25430	50.9%	68.6%	58.8%	17 994
Moyne (S)	25490	60.0%	70.9%	66.8%	16 277
Murrindindi (S)	25620	60.8%	69.6%	65.5%	13 494
Nillumbik (S)	25710	64.2%	70.9%	69.1%	62 724
Northern Grampians (S)	25810	56.4%	72.8%	66.8%	11 799
Port Phillip (C)	25900	57.7%	66.1%	61.3%	102 501
Pyrenees (S)	25990	No cases	68.2%	62.6%	6 770
Queenscliffe (B)	26080	57.4%	70.3%	64.7%	3 058
South Gippsland (S)	26170	53.4%	73.5%	65.0%	27 930
Southern Grampians (S)	26260	63.2%	73.3%	70.9%	16 145
Stonnington (C)	26350	54.5%	68.3%	60.8%	103 187
Strathbogie (S)	26430	59.1%	71.2%	66.8%	9 706
Surf Coast (S)	26490	56.2%	71.0%	65.3%	28 282
Swan Hill (RC)	26610	59.5%	72.4%	68.6%	20 867
Towong (S)	26670	57.8%	70.1%	65.2%	5 889
Unincorporated Vic	29399	No cases	70.3%	64.6%	758
Wangaratta (RC)	26700	58.5%	67.2%	61.9%	27 197
Warrnambool (C)	26730	54.8%	68.1%	60.7%	33 300
Wellington (S)	26810	58.7%	72.4%	67.1%	42 319
West Wimmera (S)	26890	61.9%	69.3%	66.3%	4 089
Whitehorse (C)	26980	53.6%	65.3%	58.1%	161 724
Whittlesea (C)	27070	60.7%	66.0%	63.3%	179 261
Wodonga (RC)	27170	50.7%	66.7%	57.1%	37 345
Wyndham (C)	27260	53.7%	76.0%	65.8%	189 618
Yarra (C)	27350	60.5%	59.4%	57.6%	83 593
Yarra Ranges (S)	27450	65.6%	71.7%	69.6%	149 538
Yarriambiack (S)	27630	54.6%	71.5%	64.3%	7 018

Cities (C), Rural Cities (RC), Boroughs (B) and Shires (S)

## Discussion

Our study, using geocoding of Victorian OHCA registry data, found significant regional variation in both the incidence of OHCA and rates of bystander CPR rates for witnessed arrests. This variation was seen across the entire state, with differences seen in neighboring communities in both metropolitan Melbourne and regional areas. Areas with high incidence and low bystander CPR were able to be identified.

Reported OHCA incidence rates are known to vary both within and across countries[3], including in previous reports from Australia[24,25]. Our data suggests some of this variation may be explained by the regions examined and in the periods of time studied. We found significant sub-State variation in OHCA incidence rates, which is consistent with reports across census tracts in the United States[26–28]. To our knowledge, ours is the first study to include rural regions with low and very low density populations (<200/km2), some of which had the highest incidence rates across the state. Our study also noted a decline in the overall incidence over the study period, which was also reported in another Australian study[29], but this decline was not consistently seen across the state. The fluctuations in incidence rates over time in our study is contrary to a previous US study[26], which found incidence rates across census tracts to be stable between two consecutive 2-year periods. However, it’s worth noting that we compared periods of different length (2 years vs 3 years), and different years (2005–2009 vs 2008–2013) to the Semple study. Similar to our study though, Semple et al. [26] did report changes in bystander CPR rates across census tracks over time [26].

Our study restricted the examination of bystander CPR rates to bystander witnessed OHCAs. We did this as witnessed arrests are strongly associated with bystander CPR[30], and rates of witnessed arrests have fluctuated over time with our region[19,31]. Increases in bystander CPR rates occurred in all but one region over the study period. This increase is difficult to explain as telephone-CPR instructions remained constant over the study period[15]. Rural regions changed from a manual system of emergency call taking to an electronic emergency call taking algorithm in 2010–11[19]. This change may have improved the recognition of OHCA in the emergency call[32] or possibly the compliance with the protocols, but does not explain the increase seen in metropolitan regions. Alternatively, it’s possible that there were changes in CPR training following the 2010 guidelines or shifts in the underlying demographics of the regions which may impact rates.

Studies based in the US and Asia have identified demographics factors that differ between low and high-risk regions. These areas tend to have specific racial compositions, and lower levels of education and income[18,33,34]. The next phase of our study intends to explore the demographic factors seen in our high risk regions and whether shifts in the population are responsible for the changes seen over time in our study. However, that these high-risk areas can change over time is an important consideration in the development and testing of community-based interventions targeting these regions. Thus interventions may choose to focus on those areas at persistently high-risk over time[26], and consider changes in relevant underlying characteristics during any evaluation period.

This study has a number of limitations. Firstly, we assigned arrests to the regions in which they occurred. This means that estimates in the regions where people commute to work may over estimate that incidence. This is particularly likely to affect the Melbourne central business district; however it’s worth noting that bystander CPR rates in this region are the highest in the state and thus unlikely to be targeted for interventions to improve bystander CPR participation. Secondly, it’s possible that the incidence of OHCA and bystander CPR rates may be correlated with population density. Thus it’s possible that estimates in rural areas may have been biased too heavily toward the mean. Although ours is a conservative approach, small-area estimation models which better explain the heterogeneity may improve estimates for data sparse regions[35].

## Conclusion

Our data supports reports of regional variation in OHCA incidence and bystander CPR rates–which in our case occurred even across a large metropolitan city and among bystander witnessed OHCAs. Identifying lower bystander CPR rates in the context of higher OHCA incidence identifies those regions where there is the greatest potential to improve survival–particularly in regions where high-risk status persists.
